# Immunomodulatory, antioxidant, and growth-promoting activities of dietary fermented *Moringa oleifera* in Nile tilapia (*Oreochromus niloticus*) with in-vivo protection against *Aeromonas hydrophila*

**DOI:** 10.1186/s12917-024-04070-3

**Published:** 2024-05-27

**Authors:** Asmaa A. M. A. Nassar, Ahlam Abd El-Aziz Gharib, Sarah Yousef Abdelgalil, Hossam M. AbdAllah, Gamal A. Elmowalid

**Affiliations:** Department of Microbiology, Faculty of Veterinary Medicine, Zagazig University, Zagazig, Egypt

**Keywords:** Fermented *Moringa oleifera*, Nile tilapia, Immunostimulation, Growth performance, Cytokines-immune related genes, *Aeromonas hydrophila*

## Abstract

**Background:**

*Moringa oleifera*, a well-known medicinal plant, has been used in aquafeed as a dietary supplement. Based on previous studies, insufficient research is available on the dietary supplementation of Nile tilapia with *M. oleifera* leaf and seed mixtures, specifically the fermented form. Therefore, this study aimed to investigate the efficacy of fermented (FMO) versus non-fermented *M. oleifera *(MO) leaf and seed mixtures on immunological parameters, antioxidant activity, growth performance, and resistance to *A. hydrophila* infection after a 30-day feeding trial on Nile tilapia.

**Methods:**

A total of 180 fingerlings were randomly divided into four groups in addition to the control group (36 fish each, in triplicate). Fish in the tested groups were fed on basal diet supplemented with MO5%, MO10%, FMO5%, and FMO10%, while those in control were fed on basal diet only. After the feeding trial, fish were challenged with *A. hydrophila*. The immunomodulatory activity of *M. oleifera* was evaluated in terms of phagocytic and lysozyme activities, immune-related cytokines and IgM gene expression. Antioxidants, and growth-promoting activities were also assessed.

**Results:**

The results revealed that fish supplemented FMO markedly in FMO10% group followed by FMO5%, exhibited significant (*P* < 0.05) improvement in the tested immunological, hepatic antioxidants, and growth performance parameters. Furthermore, the highest survival rate post-challenge with mild clinical symptoms, and the lowest *A. hydrophila* bacterial count were reported in these groups. Meanwhile, MO10%-supplementation exhibited the opposite trend.

**Conclusions:**

The study' conclusion suggests that fermented *M. oleifera* leaf and seed mixture is a promising growth-promoting and immunostimulatory feed-additive candidate for Nile tilapia and could reduce the losses caused by *A. hydrophila* infection.

## Introduction

Nile tilapia (*Oreochromis niloticus*) is one of the most intensively cultured fish species that could provide high-quality protein at an affordable cost. This industry is rapidly changing into intensification, which increases the risk of infectious disease outbreaks, along with some environmental hazards [1, 2]. Motile aeromonad septicemia (MAS) is a current threat to the tilapia farming industry. The etiological agent of this disease is usually *Aeromonas hydrophila*, which is known by its pathogenicity through the secretion of various virulence factors like hemolysins, endotoxins, enterotoxins, proteases, and lipases in immunosuppressed fish [3].

*A. hydrophila* mostly induces an ongoing inflammation that is clinically manifested by hemorrhage, ulceration, and septicemia in Nile tilapia [4]. Mortality rates due to MAS are frequently high, resulting in an overwhelming negative impact on this industry worldwide; therefore, proper control measures regarding both prevention and treatment are necessary. Competing for this and/or other aquatic pathogens depends on conventional strategies, such as antibiotics or vaccines. Antibiotic administration may be associated with the emergency of antibiotic-resistant strains that may spread to other fish and the accumulation of antibiotic residues in tissue, which possess a potential hazard to the health of human beings [5]. Additionally, vaccines are neither cost-effective nor practically applicable [6–8]. Therefore, the search for new, safe alternative strategies and products that could replace antibiotics, fortify the immune system, reduce oxidative stress, and prevent or control diseases in human and animal health is required [9]. Herbal plants are promising alternatives, not only because of their antimicrobial properties, cost-efficiency, and negligible side effects, but also because they improve growth performance and host immune status [10].

One of the herbal plants that is affordable and contains potent phytochemicals is *M. oleifera*. Interestingly, every part of *M. oleifera*, including the leaf, root, seed, and flower, is edible and contains phytochemicals that are important for human and livestock wellness. *M. oleifera* incorporation into the diets of Nile tilapia has been found to have variable biological and immunomodulatory activities [11], via improving humoral and cellular immune responses and stimulating phagocytic activity as a result of moringa-based polysaccharides and alkaloids [12]. Incorporation of *M. oleifera* leaf extract as a food-supplement in *O. niloticus* [13, 14], *Sparus aurata* [15], and *Cirrhinus mrigala* [16], diets has effectively increased the growth rate, hematological parameters, immune response, antioxidant enzyme’ activity, and disease resistance. Despite maintaining fish health in an optimum condition, in cases of infection or toxicity, low concentrations of *M. oleifera* leaf powder or its extract supplementation (0.5, 1, and 1.5%) were unable to promote fish growth performance [13, 14], and the most enhanced growth performance was achieved at 5% [17]. However, the anti-nutritional elements of moringa, like saponins and tannins, resulted in adverse effects regarding growth performance, antioxidant activities, and serum biochemistry in catfish (*Pangasius bocourti*) and *O. niloticus* when moringa was supplemented at high levels [18–21]. In order to avoid the risk of immunosuppression and growth retardation, one should consider the dose that subsequently affects the quantity of toxic compounds present in raw materials and should find effective ways of utilizing the beneficial effects of these raw natural products when incorporated into animal food [22, 23]. Fermentation is a biochemical process that helps break down large organic molecules into simpler ones. The microbial or enzymatic fermentation of plant ingredients leads to desirable biochemical changes responsible for the significant physical and biochemical modification of the fermented plants, which makes them superior to non-fermented ones in terms of organoleptic and probiotic properties, the production of peptides, in addition to antioxidants and antimicrobial activity. It also aids in the reduction of anti-nutrients and toxins [24]. A recent study proved that multi-strain microbial fermentation improves the nutritional quality as well as the anti-oxidant activity of *M. oleifera* leaves, rendering it a functional feedstuff for use in livestock diets [25]. Fermentation has significantly increased the flavonoid and polysaccharide contents of fermented moringa, which simulated non-specific immunity and antioxidant capacity and enhanced the growth performance of juvenile gibel carp [26]. It was reported that it has selectively increased flavonoid and polysaccharide contents, while it has reduced the anti-nutrient elements including saponins, tannins, phytates and oxalates [14, 25, 26]. *M. oleifera* seeds fermentation was shown to improve its digestibility and nutritional quality, particularly with the use of fungi (*Asperigillus*, *Thermoascus crustaceus*, *Mucor racemosus* and *Saccharomyces cerevisae*) to disrupt the plant cell wall and provide energy to the intestinal microbes [24, 27]. However, to our knowledge and based on previous studies, insufficient research is available on the dietary supplementation of Nile tilapia with *M. oleifera* leaf and seed mixtures. Therefore, this study aimed to evaluate the efficacy of fermented versus non-fermented* M.* *oleifera* leaf and seed mixtures on immunological parameters, antioxidant activity, growth performance, and resistance to *A. hydrophila* infection after a 30-day feeding trial on Nile tilapia.

## Materials and methods

### Preparation of the experimental diets

*Moringa oleifera* leaves and seeds were obtained from the Scientific Society of Moringa, Dokki, Giza, Egypt. The processing of *M. oleifera* leaves was done following the method of Mishra et al. [28], while the seeds were processed according to the method of Ijarotimi et al. [29], with minor modifications. The seeds were sorted, dehulled, and oven dried at 50 °C for 10 h then ground using an electric grinder (Moulinex, Grenoble, France) and sieved through 60 mm sieve to obtain raw moringa seed powder. The powder was subsequently packed in plastic container sealed with aluminum foil until being used for diet formulation. Both *M. oleifera* leaf and seed powder were sterilized at 121ºC for 20 min and cooled to room temperature. Thereafter, the powder fermentation was made by multi-strain microbial fermentation using probiotics (*Lactobacillus acidophilus* (ATCC 4356), *Bacillus subtilis* (BEST195) and *Saccharomyces cerevisae*), obtained from the Department of Microbiology, Faculty of Veterinary Medicine, Zagazig University. The strains were preserved in glycerol at -80ºc and then were revived by streaking samples of each culture on its corresponding media. *Lactobacillus acidophilus* was cultured in De Man–Rogosa–Sharpe (MRS) agar (Thermo Scientific™, USA) at 37ºC for 16 h using anaerobic jars containing Anaerocult® A gas packs. *Bacillus subtilis* was cultured in tryptic soy agar (TSA) with polymyxin (Oxoid Australia Pty Limited ©) at 35ºC for 10 h. *Saccharomyces cerevisiae* was inoculated in yeast extract-peptone-dextrose (YPD) broth containing conical tube followed by overnight incubation in an orbital shaker at 30 °C with agitation at 200 rpm. The grown yeast cells were then streaked on a petri dish containing 2% YPD agar (Sigma-Aldrich, USA) and incubated at 30 °C until colonies showed growth. Culture suspensions of the three microbial strains were prepared and adjusted to a concentration of 10^8^ CFU/mL with sterilized physiological saline solution. The three microbial strains were used at a ratio of 1:1:1 for the inoculation of moringa’ leaf and seed powder. The optimized co-culture parameters were as follows: total inoculation size, 24%; temperature, 32 °C; fermentation time, 6 days; and initial water content, 60%.

Fermentation of seeds and leaves was carried out separately by the method described by Honghui et al. [25]. Following fermentation, fermented moringa leaf and seed samples were oven dried at 60 °C for 12 h. After that, oven dried samples were further sieved then mixed at a ratio of 1:1 (w/w) in form of fermented mixture (FMO). Raw powdered leaves and seeds were also mixed at a ratio of 1:1(w/w) and referred to as non-fermented mixture (MO). Both MO and FMO were used for diet formulation each in two different concentrations (5 and 10%).

### Fish management

The trial was experimentally designed using a completely randomized group of Nile tilapia fingerlings in agreement with the standard procedures and policies approved by the local Institutional Animal Care and Use Committee of Zagazig University in Egypt (permission number: ZU-IACUC/2/F/388/2023). The National Institutes of Health (NIH)-approved ethical guidelines were followed for the handling and use of laboratory animals during all experimental procedures. A total of apparently healthy 180 *O. niloticus* fingerlings (30 ± 0.5 g average of initial weight) were acquired from a local hatchery (Abbassa, Sharqiyah, Egypt), and then they were transported to the Fish Research Unit, Faculty of Veterinary Medicine, Zagazig University, Egypt, where the experimental study was conducted. Initially, fish were transferred and acclimatized for two weeks in fiberglass aquaria before starting the actual experiment, in which they were immersed in a potassium permanganate (2 mg/L) and NaCl 2.5% bath for three days. Each aquarium (80 × 40 × 30 cm) was filled with 60 L of chlorine-free tap water, provided with aerators, and thermostatically controlled. Water parameters were measured and kept within the specified ranges throughout the experiment, according to the American Public Health Association (APHA) [30]. During the acclimatization period, fish were fed a basal diet (no additives) which was prepared to meet the basic nutrient requirements of Nile tilapia, according to the National Research Council (NRC) [31].

### Experimental design and feeding trial

After the acclimatization period, the fish were randomly divided into five experimental fish groups (36 each, in triplicate) and were distributed in 15 aquaria (3 aquaria per group, each of 12 fish) in a 30-day feeding trial. The five groups were designated as four moringa-fed groups where the fish were fed a basal diet containing a nonfermented *M. oleifera* leaf and seed mixture at a concentration of 5% (MO5%) and 10% (MO10%) and fermented at 5% (FMO5%) and 10% (FMO10%) in addition to the control group which was fed a basic diet free from *M. oleifera* (Table 1). The five isonitrogenous and isoenergetic experimental diets were formulated to meet the dietary requirements of Nile tilapia, *O. niloticus*, based on the recommendations of the NRC [31]. Their approximate chemical composition was presented in Table 1, which was determined following the protocols of the Association of Official Agricultural Chemists [32].

**Table 1:** Ingredients and proximate composition (% on dry weight basis) of the experimental diets

Ingredients	Control	*M. oleifera* containing diets			
		**Non fermented**		**Fermented**	
		**MO5%**	**MO10%**	**FMO5%**	**FMO10%**
Yellow corn	29	27.3	23	28.8	27.3
Wheat flour	10	10	10	10	10
PBM	18.2	23	23.3	25	28
Soya oil	3.3	3	3	3	2
SBM 44%	34.8	27.5	26.5	24	18.5
Calcium carbonate	1.5	1	1	1	1
Di-calcium phosphorus	1	1	1	1	1
Salts (Nacl)	0.2	0.2	0.2	0.2	0.2
Vits & Min premix	2	2	2	2	2
MO (5%)	0	5	0	0	0
MO (10%)	0	0	10	0	0
FMO (5%)	0	0	0	5	0
FMO (10%)	0	0	0	0	10
Total /gm	100	100	100	100	100
**Proximate calculated analysis**					
Cp%	29.81	29.85	29.75	29.86	29.86
EE%	7.31	7.64	7.63	7.92	7.33
CF%	3.72	3.93	4.45	3.70	3.95
Ash%	7.77	7.69	7.82	7.83	8.16

Vit and Min Premix:each 1 kg of premix contain: vit A 550000 IU, vit D 110000 IU, vit E 11000 mg, vit K 484 mg, vit C 50 gm, vit B1 440 mg, vit B2 660 mg, vit B3 132oo mg, vit B5 1100 mg, vit B6 1045 mg, vit B9 55 mg, Choline 110,000 mg, Biotin 6.6 mg, iron 6.6 gm, copper 330 mg, Mn 1320 mg, Zn 6.6 gm, Se 44 mg, iodine 110 mg

*PBM* Poultry byproduct meal, *SBM* Soya bean meal, *CP* crude protein, *EE* energy expenditure, *CF* crude fiber

Chemical analysis of both MO and FMO mixtures was performed. Total phenolic concentration in the MO and FMO samples was determined using the Folin-Ciocalteu colorimetric method [33], and expressed as milligrams of gallic acid equivalent (GAE) per gram (g) dry weight. Total flavonoid content was analyzed using the aluminum chloride colorimetric method [34, 35], then estimated using the quercetin standard calibration curve, and the obtained results of flavonoids were expressed as micrograms of quercetin equivalent (Qu) per 1 g of dry weight. Tannin concentration was determined by using tannic acid as a reference compound, following the method described by Broadhurst et al. [36], while total saponin content (TSC) was estimated by using the modified vanillin-sulphuric acid TSC assay, a spectrophotometric method proposed by V. Le, Anh, et al. [37], and it was expressed as mg aescin equivalents per gram dry weight of powder (mg AE/g).

Fish were fed twice daily at 8:00 a.m. and 4:00 p.m., six days per week, for thirty days at a rate of 3% of their biomass. The daily feed intake was modified based on changes in fish weight every 15 days. During the 30-day feeding trial, fish were monitored at regular intervals, with records kept for any clinical symptoms, mortality, and postmortem findings.

### Sample collection post-*M*. *oleifera* feeding

#### Blood and serum samples

At the end of the feeding trial, the fish were starved for 24 h. At day 32^nd^ and directly before collecting blood samples, fish were first anesthetized with 100 mg/L benzocaine solution (Al-Nasr Pharmaceutical Chemicals Co., Egypt). Then blood samples were drawn at random from the fish's caudal vein (4 samples per group) using sterile, disposable syringes rinsed with heparin. The blood samples were divided into two portions. One portion was transferred to tubes without anticoagulant, followed by centrifugation at 3000 rpm for 10 min at 4 °C, then the serum was carefully separated, and a portion was stored at − 80 °C for analysis of the lysozyme activity, while the other portion was freshly used for the preparation of pooled homologous fish sera (used in phagocytosis assay). The other whole blood portion was transferred to tubes containing anticoagulant agent (EDTA-coated tubes) (Al-Nasr Pharmaceutical Chemicals Co., Egypt), and it was used to assess the peripheral blood mononuclear cells’ (PBMCs) phagocytic activity.

#### Liver samples

Fish liver samples (6 fish per group), collected post-the 30-day feeding trial, were used for estimation of *M. oleifera* antioxidant activity via detection of hepatic antioxidant enzymes [(super oxide dismutase, SOD) and reduced glutathione, GSH)], as well as malondialdehyde (MDA).

### In-vivo challenge with *A*. *hydrophila*

At the end of the feeding trial, the fish were rested for 24 h. Consequently, all experimental groups, in addition to part of the control (positive control), were challenged with *A. hydrophila,* while the other part of the control remained unchallenged (the negative control). The *A. hydrophila* strain (ATCC 7966) was obtained from the Department of Microbiology, Faculty of Veterinary Medicine, Zagazig University. It was identified by its 16S *rRNA* gene sequence and screened for the presence of some virulence genes, including hemolysin (*hlyA*), aerolysin (*aer*), lipase (lip), and cytotonic heat-stable enterotoxin (*ast*) [38]. Its median lethal dose (LD50 = 1.5 × 10^7^) was determined (data not shown). Ten fish per group were intraperitoneally injected with 0.2 mL of virulent *A. hydrophila* suspension 1.5 × 10^7^ CFU/mL (LD 50) [39]. During the challenge period (14 days), the fish continued to feed on the same respective feeding regimes, and clinical signs, mortalities, and PM lesions were reported. The survival rate (SR) was calculated using the following formula: (Number of surviving fish post-challenge/Number of *A. hydrophila-*injected fish) × 100 at the 14^th^ day of the bacterial challenge.

### Sample collection post-*A*. *hydrophila* challenge

#### Head kidney samples (HK)

Fish head kidney (HK) samples (*n* = 6) were randomly harvested from 6 fish per group on the 5^th^ day of the bacterial challenge. Tissue samples were kept in TRIzol (Invitrogen) and were used for analysis of the mRNA expression levels of pro-inflammatory cytokines (*TNF-α and IL-1β*), anti-inflammatory cytokine (*IL-10*), and *IgM* genes via RT-qPCR.

#### Liver and spleen samples

The freshly dead fish' liver and spleen were aseptically collected and kept frozen at -80˚C. For the still-live fish, several fish were randomly caught from the aquaria, representing each group. The fish were anesthetized, dissected and *A. hydrophila* was re-isolated from the challenged fish’ liver and spleen samples (*n* = 3 each/group), and the viable bacterial count (CFU/g) was calculated via the streak plate method.

### Immunological parameters evaluation

#### Phagocytic activity (percentage and index)

The phagocytic activity of PBMCs in the different fish groups was evaluated post-*M. oleifera* feeding following the method described by Ainsworth and Chen [40]. The peripheral blood immune cells were isolated according to the method of Waterstrat et al. [41], and their viability was determined by trypan blue exclusion assay [42]. *Candida albicans* yeast obtained from the Department of Microbiology, Faculty of Veterinary Medicine, Zagazig University, was used as a phagocytosis microbial model. It was prepared and heat-inactivated according to the method described by Wood et al. [43]. The phagocytic activity percentage (PA%) was estimated as the percentage of phagocytic cells that engulfed one or more yeast cells. The phagocytic index (PI) was determined at the same time, and it equaled the total number of engulfed yeast cells divided by the number of phagocytes with engulfed yeast [43].

#### Evaluation of the lysozyme activity

Assessment of the serum lysozyme activity was performed according to the turbido-metric assay following the methods [44] and [45], with some modifications. This assay detects lysozyme activity using 0.75 ml of lyophilized *Micrococcus lysodeikticus* cells (0.2 mg/ml PBS, pH 6.2) (Catalog Number LY0100, Sigma Aldrich, USA) with 0.25 ml of serum. Lysozyme splits the peptidoglycan of the *M. lysodeikticus* cell wall, resulting in subsequent lysis of the bacterial cells and change in the solutions’ optical density during incubation of the serum and the bacterial cells. The reaction was carried out at room temperature, and the absorbance at 450 nm was measured after 0 and 10 min using spectrophotometer (BM Co. 5010, Germany). The serum lysozyme concentrations were calculated using a calibration curve of standard lysozyme with known units' activity, and then a linear-regression equation, obtained from the calibration curve, was used to calculate the lysozyme activity of the tested sera samples. One unit of lysozyme activity in the standard was defined as the amount of lysozyme causing a decrease in absorbance of 0.001 optical density.

### Immune-related cytokines and IgM mRNA relative expressions by Real-Time PCR (RT-qPCR)

The head kidney samples were cleaned in cold PBS buffer (pH 7.2). The primer sets for the selected immune-related cytokines (*IL-1β, TNF-α, and IL-10*) and *IgM* gene expression together with the β-actin reference gene (for normalization) were designed according to the NCBI reference sequence accession number presented in Table 2. Total RNA was extracted from head kidney tissues using the easy-RED kit (17,063, iNtRON Biotechnology), according to the instructions. The QuantiTect Reverse Transcription Kit (205311, Qiagen, Germany) was used in accordance with the manufacturer’s instructions to produce cDNA from a total of 1.0 μg of RNA, and cycling was done using the Rotor-Gene Q 2 plex. Real-Time PCR System using TopReal SYBR green master mix (RT500S, Enzynomics, Korea) following the manufacturer’s instructions. The PCR cycling conditions included an initial denaturation at 95 °C for 12 min, followed by 40 cycles of denaturation at 95 °C for 20 s, annealing at 60 °C for 30 s, and extension at 72 °C for 30 s. All gene tests were done in duplicate. PCR amplification was performed under standard conditions. A melting curve analysis was performed following PCR amplification to determine the specificity of the amplified product. After RT-qPCR was conducted and the threshold cycle (Ct) values of each sample were obtained, the relative mRNA expression levels of the tested cytokines and *IgM* were normalized using the mRNA expression of a known housekeeping gene, *β actin*. Results were expressed as fold-changes [46].

**Table 2:** Designated primer sequences and target genes for SYBR green RT-qPCR

Target gene	Primer sequence	Tm	Product size (bp)	Primer efficiency	*R*^2^ value	Accession no
**β- actin**	F: GCAGGAGTACGATGAGTCCG	59.97	157	98.5	0.9962	XM_003443127.5
	R: CTCTGCGCCTGAGTTGTGTA	60.04				
**TNF-α**	F: CTGCTCCCTTCCACTCCTTG	60.04	74	101.5	0.9748	XM_013266975.3
	R: CCGCTATCTGTGAGAGGCTG	59.97				
**IL-1β**	F: TCTTAGCGCTCCACTCCTTG	59.47	91	94.5	0.9825	XM_019365844.2
	R: CTGCCTGACTGTCCTGACTC	59.75				
**IL-10**	F:.ATGAGCAGAAGGCCTGTCAC	60.04	84	97.56	0.9681	XM_013269189.3
	R: GCTCCCCAAATAGCCACACT	60.03				
**IgM**	F: GCGACAGTCACAGTTCCTCA	59.97	131	100.5	0.9924	KC708223.1
	R: TGCTGCTACCATCACTGCAA	59.96				

*F* forward, *R* reverse, *bp* base pair

### Determination of hepatic antioxidants activity

The fish' liver samples, collected from Nile tilapia in all tested groups, were homogenized in ice-cold 50 mM sodium phosphate buffer (pH 7) containing 0.1 mM ethylene diamine tetra acetic acid (EDTA) to obtain 10% (W/V) homogenate. The homogenates were centrifuged at 4 000 g for 30 min at 4 °C, and the supernatant was aliquoted and kept at − 20 °C for estimation of oxidative stress’ marker (MDA) and antioxidant enzymes (SOD and GSH).

### Determination of super oxide dismutase (SOD)

Hepatic SOD was measured according to the method described by Nishikimi et al. [47], a colorimetric method that detects SOD in the liver sample depending on the ability of the enzyme (SOD) to inhibit phenazine methosulphate (Catalog No.: SD 25 21, Bio-diagnostics Co., Egypt) PMS-mediated reduction of Nitro blue Tetrazolium (NBT) dye, which then reduces NBT. The change in absorbance at 560 nm, related to the amount of NBT present, was then spectrophotometerically measured for 5 min, which is directly proportional to the amount of SOD present in the sample.

### Determination of reduced glutathione (GSH)

The reduced glutathione (GSH) was detected in the liver sample according to the colorimetric method reported in Beutler et al. [48], which depends on the ability of GSH to reduce a chromogen (DNTB) (Catalog No: GR 25 11, Bio-diagnostics Co., Egypt) with subsequent production of a yellow compound (reduced chromogen) when present in an acidic medium. The resultant, yellow-colored reduced chromogen was directly proportional to GSH concentration, and its absorbance was measured spectrophotometerically at 405 nm. SOD levels in each fish group were expressed as U per gram of hepatic tissue, while GSH activity was expressed as mg per gram of hepatic tissue.

### Determination of Malondialdehyde (MDA)

The liver homogenates, collected and prepared as described in SOD and GSH, were used to assess MDA in the different fish groups. Hepatic MDA was measured according to the colorimetric method reported in previous studies [49, 50]. The assay depends on the principle that Thio barbituric acid (TBA) (Catalog No: MD 25 29, Bio-diagnostics Co., Egypt), a chromogen, reacts with MDA, present in liver sample, in acidic medium at temperature of 95 °C for 30 min to form TBA reactive product of a pink color whose concentration was directly proportional to the MDA concentration in the sample. The absorbance of the resultant pink product was measured at 534 nm via a thermostated spectrophotometer (VAR-CARY-400, Canada). The MDA levels in each group were expressed as nmol/g tissue.

### Growth performance

At the end of the 30-day feeding trial, fish in all groups were fasted for 24 h and anesthetized with 95 mg/L clove oil water bath. Five fish were randomly collected from the aquaria of each experimental group, and each fish was individually weighed on a digital meter to evaluate their final weight. Then, the mean was calculated. In order to evaluate the growth-promoting activity of *M. oleifera, the* fish’s weight gain rate (WGR), specific growth rate (SGR), average daily gain (ADG), and feed efficiency (FE) were determined using the formulas reported in Zhang et al. [26].

### Statistical analysis

The data were edited in Microsoft Excel (Microsoft Corporation, Redmond, WA, USA). A Shapiro–Wilk test was conducted in order to check for normality, as described by Razali and Wah Data [51]. The significant effects of the treatments were examined according to the one-way ANOVA [52], with the level of significance set at α = 0.05. Results were expressed as means ± SE. Turkey’s’ test was used to perform pairwise comparisons between means in cases where a significant effect was detected. The significant differences between survival rates were examined according to the chi-square test. The statistical significance between means was set at a *p*-value less than 0.05. Figures were fitted by the GraphPad Prism software 9.0 (GraphPad, USA).

## Results

### Chemical analysis

#### Effect of solid-state fermentation on total flavonoid and phenolic content

In this study, the concentration of total phenolics and flavonoids was evaluated in both fermented moringa leaf and seed mixtures (FMO) and nonfermented moringa (MO). The results revealed that fermentation has significantly increased (*P* < 0.05) the concentration of phenolics and flavonoids in the FMO mixture compared to their concentration in the nonfermented MO mixture, as shown in Table 3.

**Table 3:** Bioactive and anti-nutrient composition of non-fermented and/or fermented moringa leaf and seed mixture

Ingredient	MO mixture	FMO mixture
Total flavonoids [(mg (QuE)/gm dw)]	9.5^a^ ± 0.031	17.8^b^ ± 0.15
Total phenolics [(mg (gal)/gm dw)]	17^b^ ± 0.112	26.23^c^ ± 0.112
Total saponins (mg AE/gm)	2.9^b^ ± 0.020	2.4^b^ ± 0.018
Total tannins (TanE/gm)	3.9^a^ ± 0.01	1.62^c^ ± 0.005

Each value in the Table was obtained by calculating the average of the three experiments (Mean ± S.E). The superscript letters indicated statistically significant differences, with *P* < 0.05. MO: moringa leaf and seed mixture (1:1) (w/w); FMO: fermented moringa leaf and seed mixture (1:1) (w/w).

*CP* crude protein, *EE* energy expenditure, *CF* crude fiber

#### Effect of solid-state fermentation on anti-nutritional factors

The contents of the two major *M. oleifera* anti-nutrients saponins, as well as the tannins were estimated. As shown in Table 3, both the tannins and saponins levels were significantly decreased (*P* < 0.05) in the fermented mixture, and their concentrations were lower than those of the nonfermented moringa leaf and seed mixture (Table 3).

#### Chemical composition of the fermented and nonfermented *M*. *oleifera* leaves and seeds

The approximate chemical composition of nonfermented (MLs and MSs) and fermented (FMLs and FMSs) moringa was estimated (Table 4). The results indicated that the concentrations of the estimated elements were significantly increased after fermentation.

**Table 4:** Proximate chemical composition of moringa leaves and moringa seeds

Ingredient	MLs	FMLs	MSs	FMSs
CP	28.28^a^ ± 0.10	30.19^a^ ± 0.10	29.9^a^ ± 0.15	35.33^a^ ± 0.10
CF	28.6^c^ ± 0.12	24.4^c^ ± 0.10	8.8^d^ ± 0.04	6.57^b^ ± 0.10
EE	3.5^b^ ± 0.04	4.42^b^ ± 0.12	19.5^c^ ± 0.14	23.6^a^ ± 0.10
Ash	8.05^c^ ± 0.44	10.13^b^ ± 0.32	5.56^a^ ± 0.28	4.68^b^ ± 0.15

Each value in the table was obtained by calculating the average of the three experiments (Mean ± S.E)

The superscript letters indicated statistically significant differences, with *P* < 0.05

*MLs M. oliefera* leaves, *FMLs* fermented *M. oleifera* leaves, *MSs M. oleifera* seeds, *FMSs* fermented* M. olifera* seeds, *CP* crude protein, *EE* energy expenditure, *CF* crude fiber

In the present study, fermented and nonfermented *M. oleifera* formulas, each in two different concentrations, were tested as functional feed additives to exert immunostimulatory effects in terms of phagocytosis and lysozyme activities post-feeding, as well as to induce modulation of immune-related cytokines, and *IgM* gene expression in Nile tilapia post challenge.

#### Fermented *M*. *oleifera* enhanced the phagocytic activity

**Fig. 1:**
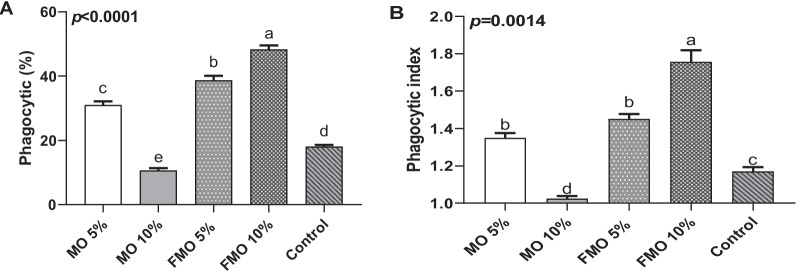
Phagocytosis percentage (**A**) and index (**B**) in the different experimental fish groups. Blood was collected from four fish in each group; PBMCs were isolated, and phagocytic percent (**A**) and index (**B**) were evaluated. Values are the means ±SD of four fish per experimental group. Means in the same raw with different superscripts are significantly different (*p* <.0001)

Fish dietary supplementation with fermented *M. oleifera* showed a remarkable and significant impact on phagocytic cell activity (*P* < 0.0001). When considering phagocytic activity (%) and phagocytic index, the group treated with FMO 10% exhibited the highest levels, followed by the FMO 5% group, compared to the control and the other treated groups (*P* < 0.0001; Fig. 1a and b). In contrast, feeding nonfermented MO10% significantly (*P* < 0.0001) reduced both phagocytic activities and phagocytic indices in the tested fish compared to the control and other moringa-supplemented groups.

#### Improvement of lysozyme activity

**Fig. 2:**
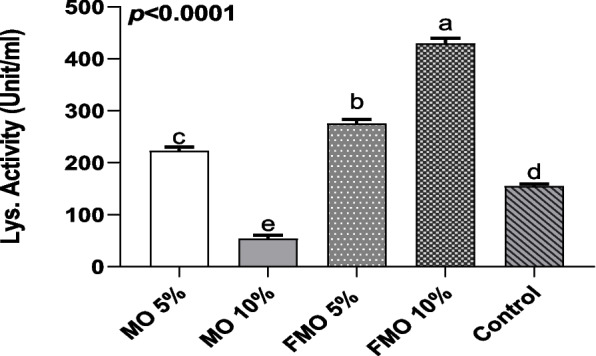
Serum lysozyme activity in the fish supplied with dietary *M*. *oleifera* and the control. After the feeding trial, lysozyme activity was estimated in the different experimental fish groups, and the reported results are the average results of four fish from each fish group. Values are the means ± SD of four fish per experimental group. Means in the same raw with different superscripts are significantly different (*P* < .0001)

To evaluate the immunomodulatory activity* of M. oleifera*, lysozyme activity against *M. lysodeikticus* was assessed after the 30-day feeding trial, as shown in Fig. 2. Notably, significant differences (*P* < 0.0001) were observed between the groups fed on nonfermented (MO5% and MO10%) and fermented *M. oleifera (*FMO5% and FMO 10%) feeds regarding lysozyme activity. The optimum inclusion rate of dietary *M. oleifera* required for serum lysozyme activity was observed in fish groups receiving FMO10%, followed by those supplemented with FMO5% and MO5% (Fig. 2). Meanwhile, the lowest level of serum lysozyme was detected in those who received MO10% compared to the control and the other fish groups.

### Modulation of head kidney-immune-related cytokines and *IgM* gene expression levels

**Fig. 3:**
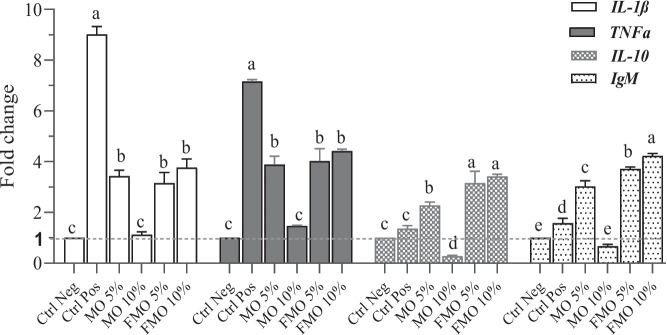
mRNA fold-change expression levels of *TNF-α, IL-1β, IL-10****,*** and *IgM* of *O. niloticus *at day five post-challenge with *A. hydrophila *in the head kidneys of the different fish groups. Data are expressed as mean ± SEM (6 samples/group) Values (*p* < 0.0001). Ctrl Neg: negative control (non-infected); Ctrl Pos: positive control (infected)

The gene fold-changes of immune-related cytokines (*IL1β, TNF-α, and IL-10*) and *IgM* detected in the different experimental groups post-challenge with *A. hydrophila* are expressed in Fig. 3. On the 5^th^ day of the bacterial challenge, the relative mRNA expression fold-changes of pro-inflammatory cytokines (*IL-1β*, *TNF-α)* and anti-inflammatory cytokines* (IL-10),* in addition to *IgM,* varied significantly among the experimental groups. The pro-inflammatory cytokines were less upregulated in *M. oleifera* supplemented groups compared to those in the positive control, where these pro-inflammatory cytokines were dramatically upregulated. The m RNA expression values of pro-inflammatory cytokines were higher in fish groups fed diets supplemented with FMO10%, followed by FMO5%, and MO5% respectively, along with a considerable upregulation of *IL10* and *IgM* in a dose dependent trend with greatest effects to FMO 10%. Meanwhile, the MO10% group showed a modest upregulation of pro-inflammatory cytokines with a correlated downregulation of anti-inflammatory (*IL-10*) cytokines whose expression level was lower than that of the positive control. A significant decrease in the expression level of *IgM* was also detected in the MO10% group compared to the positive control and other moringa fed groups.

### Hepatic antioxidants activities modulation

**Fig. 4:**
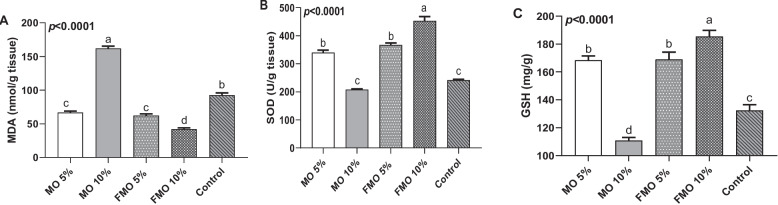
Effect of experimental diets on redox status, including lipid peroxidase (MDA; A), superoxide dismutase (SOD; B), and glutathione (GSH; C). Data are expressed as mean ± SEM (6 samples/group) Values (*p* < 0.0001)

The activities of SOD and GSH in the hepatic tissues of the tested fish groups are shown in Fig. 4b and c. Both SOD (Fig. 4b) and GSH (Fig. 4c) activities were significantly (*P* < 0.0001) high in the FMO10% and FMO5% groups, followed by MO5%, while being significantly reduced in the fish received MO10% compared to the control. In terms of lipid peroxidation, the MO10%-treated group exhibited the highest level of MDA (Fig. 4a); however, it significantly decreased in fermented moringa-supplemented groups, particularly in the FMO10% group.

### Growth performance and feed efficacy in Nile tilapia post *M*. *oleifera* feeding

**Fig. 5:**
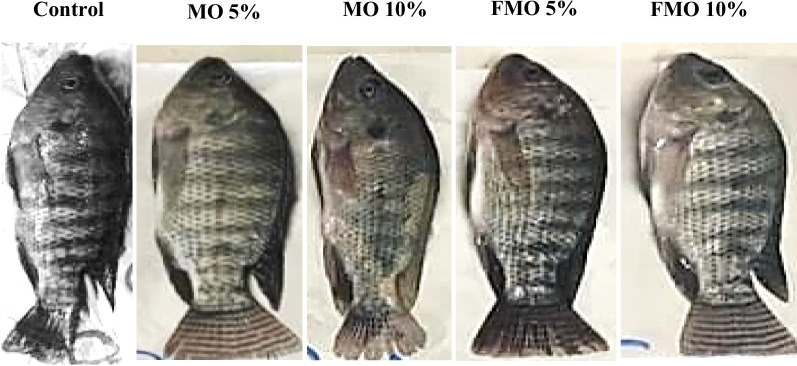
Morphological and growth appearance of fish fed on fermented and nonfermented *M. oleifera* leaf and seed mixtures post-challenge with *A.* *hydrophila*

The fish were supplemented with nonfermented (MO 5 and 10%) or fermented (FMO 5 and 10%) *M. oleifera* for 30 days, and then photographs were taken of randomly selected fish in each group. Fish supplemented with fermented *M. oleifera* and nonfermented (MO 5%) showed significant enhancement in growth performance (Fig. 5). The present findings demonstrated that Nile tilapia supplementation with *M. oleifera* leaf and seed mixtures optimized growth parameters (WGR, ADG, and SGR) in a 30-day feeding trial. The results of growth performance and food utilization in Nile tilapia-fed diets containing different levels and formulas of *M. oleifera* leaf and seed mixtures are summarized in Table 5. The results revealed that there was no significant difference (*P* < 0.0001) between fish receiving nonfermented *M.* oleifera at a level of 10% (MO10%) and the control group receiving basal diet in the values of final weight, weight gain rate, average daily gain, specific growth rate, and feed efficiency percentage. Meanwhile, a highly significant increase in the same growth performance parameters was observed in fish fed fermented (FMO 10%) and non-fermented (MO5%) *M. oleifera* leaf and seed mixtures compared to the control group, as shown in Table 4 and Fig. 5.

**Table 5:** Growth performance, feed utilization of *M*. *oleifera* (nonfermented or fermented) leaf and seed mixture-supplemented fish

Item	Control	MO5%	MO10%	FMO5%	FMO10%	*p*-value
IBW (g)	30.00 ± 0.50	30.00 ± 0.50	30.00 ± 0.50	30.00 ± 0.50	30.00 ± 0.50	1.00
FBW (g)	33.70 ± 2.36^**c**^	44.40 ± 1.50^**a**^	32.20 ± 0.81^**c**^	38.40 ± 0.76^**b**^	45.00 ± 0.23^**a**^	< .0001
ADG (g)	0.14 ± 0.07^**c**^	0.49 ± 0.06^**a**^	0.11 ± 0.01^**c**^	0.29 ± 0.01^**b**^	0.51 ± 0.01^**a**^	< .0001
WGR (%)	13.94 ± 8.04^**c**^	50.46 ± 7.37^**a**^	11.40 ± 1.45^**c**^	29.73 ± 1.24^**b**^	52.30 ± 1.41^**a**^	< .0001
SGR (% d^−1^)	0.43 ± 0.20^**c**^	1.35 ± 0.16^**a**^	0.35 ± 0.04^**c**^	0.87 ± 0.03^**b**^	1.40 ± 0.03^**a**^	< .0001
FE (%)	16.08 ± 0.01^**c**^	62.59 ± 0.03^**a**^	9.56 ± 0.01^**d**^	36.51 ± 0.02^**b**^	65.20 ± 0.04^**a**^	< 0.0001

At the end of the feeding trial, 15 fish from each experimental group were directly challenged with virulent *A. hydrophila.* Consequently, 15 fish from each group were challenged with virulent *A. hydrophila,* and the survival rate, SR (percent in the parenthesis), was calculated

Values are the means ± SD of five fish per experimental group. The means in the same raw with different superscripts are significantly different. (*p* < .0001)

*IBW* initial body weight, *FBW* final body weight, *ADG* average daily gain, *WGR* weight gain rate, *FE* feed efficiency, were calculated

### Clinical signs in the different fish groups post challenge

**Fig. 6:**
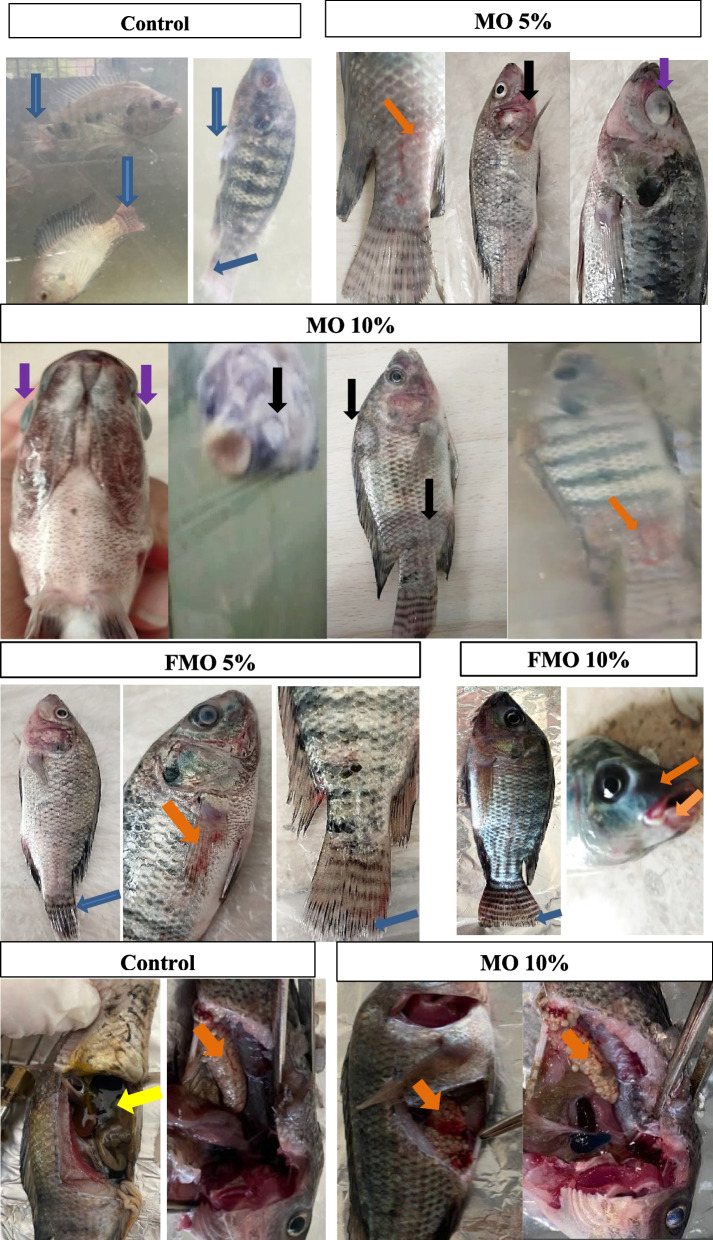
Clinical symptoms and post-mortem pathological lesions in the different Nile tilapia groups post-challenge with *A. hydrophila*. Blue arrow: tail and fin erosions. Orange arrow: hemorrhage or hyperemia. Black arrow: ulcers. Violet arrow: exophthalmia. Yellow arrow: ascites

After challenging Nile tilapia with *A. hydrophila*, different clinical symptoms were observed. Twenty-four h post-induced infection, all fish decreased their feeding rate and exhibited erratic swimming behavior, which continued for 72 h. The clinically.

inspected positive control and* M. oleifera-*fed fish exhibited many pathological symptoms of variable intensity, including pale gills, hemorrhages, hyperemia on the skin, scale detachment, ulcers, erosion of the tail, and fins. Some fish exhibited unilateral or bilateral exophthalmia. Ascites, congestion, and petechial hemorrhage on internal organs were also detected on PM analysis, as summarized in Table 6, and Fig. 6. The MO10%-treated group exhibited the most intense pathological changes in external and internal organs, which were even more severe than the symptoms observed in the positive control group. Moderate clinical symptoms and/or PM lesions were observed in the MO5% group, while mild erosions of the tail and fins were observed in fish supplemented with FMO10% and FMO5%.

**Table 6:** Clinical symptoms and post-mortem pathological features of the different Nile tilapia groups post-challenge with *A. hydrophila*

Item	Ctrl Neg	MO 5%	MO 10%	FMO 5%	FMO 10%
Skin hemorrhage	**+ + **	**+ **	**+ + + + **	**+ **	**-**
Scale’s detachment	**+ + + **	**+ **	**+ + + + **	**-**	**-**
Ulcers	**+ + **		**+ + + **	**-**	**-**
Erosion of tail and fins	**+ + + **	**+ **	**+ + + + **	**+ **	**+ **
Uni or bi- lateral exophthalmia	**-**	**+ **	**+ + **	**-**	**-**
Congestion of internal organs	**+ **	**+ **	**+ **	**+ **	**+ **
Petechial hemorrhage of internal organs	**+ + + **	**+ **	**+ + + **	**-**	**-**

### Fish protection and survival rate post-challengewith *A*. *hydrophila*

**Fig. 7:**
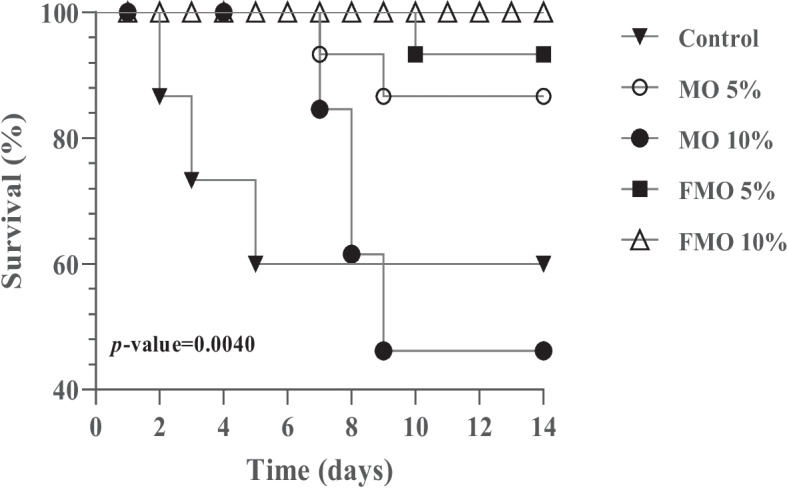
Survival rates of fish (*n* = 15 in each group) challenged with virulent *A. hydrophila* for 14 days and supplemented with fermented and nonfermented *M. oleifera* leaf and seed mixture

The results regarding the survival rate of fish supplemented with different levels and formulas of *M. oleifera* leaf and seed mixtures are displayed in Fig. 7. The results revealed highly significant growth-promoting effects on tilapia as a result of fermented moringa dietary supplementation. A significantly higher survival rate (*P* = 0.0040) was obtained in the fish fed FMO10% (100%), followed by those received FMO5% (93%), compared to the control group (60.0%). The lowest survival rate (53%) was reported in the fish fed on non-fermented moringa (MO10%).

### *A*. *hydrophila* counts (CFU/g) of fish’ liver and spleen post-challenge

**Fig. 8:**
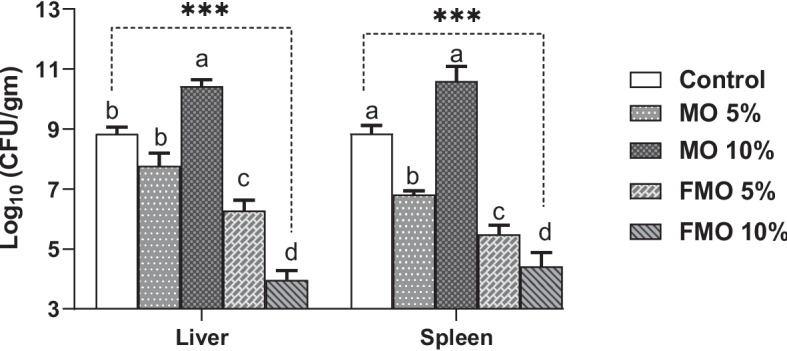
Bacteriological colony count (CFU/gm) of the challenged fish’ liver and spleen. ***p < 0.0001. Values are means ± SD (*n* = 3 liver and/or spleen samples per group). Means in the same raw with different superscripts are significantly different (*p* < .0001)

To determine the mortality cause and to evaluate the fish' capacity to control *A. hydrophila* infection, the *A. hydrophila* bacteria were re-isolated, and their viable count (CFU/g) was determined in the challenged fish’ liver and spleen. It was observed that fermented* M. oleifera* dietary supplementation had a highly significant impact on the bacteriological count isolated from the liver and spleen of the challenged fish (*p* < 0.0001). The MO10% treated groups exhibited significantly higher bacteriological counts in the liver and/or spleen compared to other treated groups, as well as the control (Fig. 8). Fish groups received fermented moringa (FMO 5 and 10%), followed by those received MO5%, manifested a low bacterial count in their liver and spleen compared to MO10% and the control groups.

## Discussion

The improvement of disease resistance and the enhancement of fish immunity have become urgent needs in healthy aquaculture. Developing efficient preventive immunomodulators in aquaculture as substitutes for vaccinations and chemotherapy has been the focus of numerous studies. In the current study, dietary* M. oleifera* leaf and seed mixture immunomodulatory, growth-promoting, and antioxidant activities were evaluated, with subsequent challenges with *A. hydrophila* infection.

Findings in the current study indicated that dietary supplementation with FMO10% significantly enhanced phagocytic cell activity, as evidenced by the increase in both phagocytic percentage and phagocytic index. In a recent study, moringa dietary supplementation was found to increase leucocyte differentiation levels of effective monocytes, neutrophils, eosinophils, and basophils, as identified by the findings of Helmiati and Isnanset [53]. Our results also agreed with those of Mansour et al. [15], whose results revealed an improvement in head kidney leucocyte phagocytosis in addition to serum humoral components, including lysozyme activity and IgM level, with *M. oleifera* leaf dietary inclusion, especially at the 5% level. The elevated level of serum lysozyme in the fish groups received FMO10%, FMO5%, and MO5% indicates a developed protective mechanism in fish as it is considered a key enzyme in the innate immune response, having lytic and opsonic activity against both Gram-positive bacteria and Gram-negative bacteria in addition to phagocytosis and complement system activation [54].

The immunostimulatory activity, as evidenced by the phagocytic and lysosomal-enhancing activity of *M. oleifera,* may be correlated with the richness of *M. oleifera,* with abundant contents of many biologically active phytochemicals such as polyphenols, volatile oils, vitamins such as vitamin E, A, and C, phenolic acids, and flavonoids, which exist at higher concentrations in the fermented moringa [55–59].

It also may be attributed to the immunomodulatory compounds of *M. oleifera,* such as *M. oleifera*-based polysaccharides (MOPs), specifically MOP-2, that were reported to serve immuno-regulatory activity via activation and proliferation of macrophages as well as increasing phagocytic activity [60]. Dietary alkaloids found in *M. oleifera* can also enhance humoral and cellular immune functions by stimulating the phagocytic activity of macrophages with subsequent stimulation of lysosome activity and respiratory burst activity [12]. Furthermore, *M. oleifera* seeds were found to enhance the immune response and prevent diseases, as seed-soluble fiber and lectins were found to stimulate the proliferation of murine macrophages, in addition to T-cell activation [61]. Our results clearly indicated that the moringa' immunomodulation in Nile tilapia was dose-dependent and affected by fermentation. Higher *M. oleifera* concentrations have been previously found to decrease phagocytic activity [62]. This agrees with our results, as increased levels of *M. oleifera* beyond the 5% level have a negative effect on the overall health status, producing an immunosuppressive effect on the host, as observed in the MO 10% treated group. These findings concurred with those previously reported by Abidin et al. [63], who found that shrimp fed moringa extract incorporated at 2.5 g/kg showed the highest phagocytic activity compared to those received moringa at a concentration of 5.0 g/kg. In another study by Mahajan and Mehta [64], regarding the immunomodulatory effect of *M. oleifera* seed extract, the immunosuppressive activity of the ethanolic extract of the seeds was dose-dependent as at higher doses it induced a reduction in circulatory leukocyte and splenocyte counts and caused a down-regulation of macrophage phagocytosis. Anti-nutrient elements detected in moringa, such as oxalate, resulted in an impairment of the immune response, as evidenced by a subsequent decrease in monocyte metabolism and chemoattractant protein (MCP-1) secretion, which is responsible for the recruitment of monocytes and macrophages to tissues in order to remove pathogens [65]. Therefore, when using plant materials in animal feed, we should take into account the toxic chemicals contained in raw materials for animal feed as by increasing the herbal dose the amount of toxic materials subsequently increases and it that could be a strong potential cause of the resultant immune suppression occurred in MO10% group.

Fermentation results in an increase in iso-flavones, which are proven disease-protective components, and total flavonoid aglycones that become more easily and rapidly absorbed in the intestine after enzymatic digestion and bacterial fermentation [66, 67]. The increase in the concentration and bioavailability of the essential nutrients as well as the bioactive phytochemicals, in addition to the decrease in the content of the anti-nutritional elements, and furthermore, the presence of antimicrobial plant-derived peptides post-enzymatic hydrolysis and multi-strain microbial fermentation of *M. oleifera* [68], may explain the significant improvement in the immune response and disease resistance observed in fish supplemented with fermented *M. oleifera* leaf and seed mixture.

The current study aimed to investigate the interactions between blood immune parameters (phagocytic activity, serum lysozyme) and the mRNA expression of immune-related genes (*TNF-α, IL1β, IL-10,* and* IgM*) and their protective effect on Nile tilapia in response to *A. hydrophila* infection and also to indicate the immunomodulatory effect of *M. oleifera* regarding the release of both pro-and anti-inflammatory cytokines in fish post infection in order to evaluate its potential in protection against systemic inflammatory response syndrome (SIRS) and septic shock caused by virulent *A. hydrophila*. The TNF-α and interleukin-1β serve to initiate host defense against microbial invasion in the form of inflammatory response. The wisely induced up-regulation of these pro-inflammatory cytokines (*p* < 0.05) in Nile tilapia fed on fermented (FMO 5 and 10%) and nonfermented (MO 5%) *M. oleifera* leaf and seed mixtures may be attributed to the immunomodulatory compounds of *M. oleifera,* such as MOP-2, that was reported to serve immuno-regulatory activity on macrophages via enhancing the secretion of various pro-inflammatory cytokines, such as TNF-α and IL6 [60], these MOPs were further increased following moringa fermentation [26], which may explain the increasing trend of these cytokines in FMO- supplemented groups compared to MO- supplemented groups. On the other hand, the pro-inflammatory cytokines were dramatically upregulated post *A. hydrophila* infection in the positive control which might be associated with the activation of the toll-like receptors (TLRs) pathway by the lipopolysaccharide content of *A. hydrophila* cell wall which in turn activated NF-κβ that induced the expression of pro-inflammatory cytokines through a MyD88-independent pathway [69]. This interesting modulation of pro-inflammatory cytokine’ response in the above-mentioned groups compared to the positive control is possibly associated with the high quercetin and isothiocyanates contents in moringa known for their properties in alleviating the inflammation, which may have regulated the expression of these pro-inflammatory cytokines by influencing TLRs through NF-κβ signaling pathway [70, 71]. We expect that those investigated pro-inflammatory cytokines stimulated the recruitment of macrophages and neutrophils, resulting in their migration and activation, which are necessary for the initiation of the immune response against bacterial invasion via microorganism recognition, antigen presentation, and stimulation of the protective immune response in the above-mentioned groups [72]. However, excess inflammation can give rise to systemic disturbances harmful to the host, so regulation of the inflammatory response is important in order to shift body mechanisms towards tissue repair and prevent further tissue damage from ongoing inflammation. Interestingly, the immunomodulatory compounds of low dose (MO5%) and fermented *M. oleifera* stimulated the development of optimum parallel anti-inflammatory mechanism via production of IL-10. Interleukin-10 (IL-10) not only functions as an anti-inflammatory cytokine that optimizes and prevents an overwhelming inflammatory response, but also maintains the balance between pathogen clearance and immunopathology against bacterial infection [73], but also performs stimulatory effects on immune cells. It mainly improves the survival rate of B cells and promotes the proliferation and differentiation of activated B cells into plasma cells. Thus, it is critical for antibody production, controlling disease progression, and resolution of the host inflammatory response. Additionally, dysregulation of IL-10 is associated with enhanced immunopathology in response to infection [74, 75]. In this study, the inflammatory response in FMO10%, FMO5% and MO5% groups was regulated by the optimal release of IL-10, whose expression levels were higher in these groups, compared to the MO10% and the positive control group which agrees with the results of previous studies [76, 77]. Meanwhile, the excessive upregulation (positive control) as well as the modest upregulation of pro-inflammatory cytokines in MO10% group may have affected the release of IL-10 and led to the resultant inadequate upregulation and the down regulation of IL-10 observed in positive control and MO10% groups respectively. Furthermore, this unregulated inflammatory response could have led to non-resolving inflammation, as explained by Nathan and Ding [78].

In our findings, IgM expression levels were high in Nile tilapia supplemented with FMO and MO 5%. IgM contributes to both innate and adaptive immunity in fish. It is considered a potent activator of the complement system, which both lyses and opsonizes pathogens. It also mediates the agglutination and opsonization of pathogens for the enhancement of phagocytosis and the removal of these pathogens [79]. The increased expression of IgM can be explained by the upregulation of IL-10, as aforementioned. Our findings ascertain the previous studies that discussed the role of IL-10 against invading pathogens and its promoting effect on IgM production in Nile tilapia and carp [80, 81]. It can also be attributed to the effect of probiotics used in fermentation, as it has been proven that probiotics can stimulate antibody production in *Oncorhynchus* *mykiss* [82], and in different fish species* fed* diets containing *B. subtilis* [83]*. Saccharomyces cerevisiae* has also been found to be effective in increasing the serum immunoglobulin IgM level of seabream through dietary supplementation [84]. Our results were consistent with those of previous researchers [26, 85], who revealed that IgM in addition to lysozyme levels were significantly increased in fermented *M. oleifera-*treated groups and in groups supplemented with MO5%, respectively. Our results also revealed that Nile tilapia survival rates post-challenge were significantly high in the FMO5% and 10% groups, followed by the MO5% groups. The increased survival rate of Nile tilapia indicated that dietary *M. oleifera* leaf and seed mixture, when supplied at low dose or in a fermented form, can increase fish resistance to bacterial invasion through its immunostimulatory activity, as evidenced by the increase in phagocytic and lysozyme activities and regulation of immune-related cytokines in addition to IgM, all of these stimulated protective mechanisms resulted in an enhanced immune response against *A. hydrophila* infection and a subsequent decrease of bacterial load in the fish internal organs which is indicated by the low bacterial count isolated from fish in these groups compared to those in the positive control group. Meanwhile, the immunosuppression indicated by the decreased phagocytic and lysozomal activities, along with the improperly activated inflammatory response, fish in the MO10% group were not able to fight against *A*.* hydrophila*, with the subsequent development of severe pathological lesions on them, a high bacterial count isolated from their organs, and eventually the low survival rate of fish in this group.

An indication of enhanced growth performance is the improvement in final body weight, weight gain rate, specific growth rate, and feed efficacy. Our results indicated that fermented (FMO5% and 10%) and nonfermented (MO5%) *M. oleifera* leaf and seed mixtures resulted in significant growth performance. Conversely, feeding nonfermented moringa at a high concentration (MO10%) did not show any significant effect on fish growth. The resultant growth promotion in the fish that received fermented moringa may be related to the fact that fermentation breaks the leaf and seed cell walls, releasing phenolic compounds known for their nutrient availability and growth-enhancing activities, which support our findings and other data reported [55, 86]. In addition, fermentation was previously found to be an effective tool to remove the anti-nutritional factors (tannins, phytates, oxalate and saponins), which interfere with several physiological functions as saponin was found to have a negative effect on the biological membranes that acts as a surface-active component leading to an increase in permeability of the intestinal mucosal cells and rendering transportation of active nutrients [87], while phytate reduces the bioavailability of minerals and reduces the protein digestibility through formation of phytic acid–protein complexes which inhibit the absorption of nutrients [88], these findings could justify the stunting growth of fish in MO10% group. It has been reported that African catfish, *Clarias gariepinus*, when supplemented with moringa leaf extract at levels > 10%, showed no improvement in growth performance compared to the control [89]. In a similar study by Richter et al. and Puycha et al. [18, 21], moringa leaves were used up to 15% of dietary protein in the diet of *Oreochromis niloticus* and/or Bocourti’s catfish (*Pangasius bocourti*) without any significant changes in fish weight or growth performance.

The level of antioxidant enzymes (GSH-SOD) was highly increased in the liver of fish fed with FMO10% and 5%, moderately in MO5%. However, it showed a significant decrease in the MO10% supplemented group compared to the control, with a subsequent increase in MDA level, a lipid peroxidation product that served as a marker of oxidative stress. supporting the results of many authors [26, 90–92]. In addition to Wu et al. [93], who found that myrtle flavonoids increased SOD and glutathione activities and reduced MDA content in the serum. This enhanced antioxidant activity can be attributed to the fact that* M. oleifera* contains natural antioxidants, such as flavonoids and other phenolic compounds.* M. oleifera* is known to have more than 40 natural antioxidant compounds that have been used to treat oxidative stress due to their effect on eradicating free radicals [94]. Isoquercetin is recorded to have the highest anti-oxidative activity, and it acts by both inhibiting free radical production and increasing the expression of antioxidant enzymes such as SOD and glutathione [95, 96]. In addition, it was proven that fermentation of the phenolic compounds in *M. oleifera* leaves into short-chain fatty acids plays an important role in modulating the gut microbiota, leading to a more balanced microbiome with subsequent enhancements in anti-oxidant activity and overall immunity [86, 97]. In addition, it was proven that fermentation of the phenolic compounds present in *M. oleifera* leaves into short-chain fatty acids plays an important role in modulating the gut microbiota, leading to a more balanced microbiome with subsequent enhancements in anti-oxidant activity and overall immunity [98]. It also changes the type and volume of active ingredients in the substrate, and the resulting small bioactive glycosides have stronger antioxidant activities [99]. The depressed anti-oxidant activity observed in the MO10% group can be attributed to the effect of excess oxalates [100], when moringa was included at a high concentration, as it was proven that oxalate loading resulted in reduced glutathione levels and increased reactive oxygen species (ROS) generation, suggesting impaired anti-oxidant activity too [65].

## Conclusions

This study confirmed that immune-stimulatory responses, antioxidants, growth promotion, and resistance to *A. hydrophila* infection were differently affected according to moringa concentration and formulation in the Nile tilapia diet. *M. oleifera* leaf and seed mixture, supplemented at low concentrations (MO 5%) or fermented, with the greatest effect of FMO 10%, showed a potent immune-stimulatory effect on Nile tilapia fish as it enhanced the phagocytic and lysozyme activities, regulated the expression of immune-related cytokines and IgM genes, potentiated the liver's antioxidant enzyme production, improved fish’s growth and overall health status, and increased Nile tilapia's protection against *A. hydrophila.* In conclusion, our results suggest that multi-strain microbially fermented *M*. *oleifera* can be utilized as a feed additive for aquaculture to reduce the application of antimicrobials in fish farming.

## Data Availability

All data generated or analyzed during this study are included in this published article.
